# A Comparison of Different Methods to Estimate the Effective Spatial Resolution of FO-DTS Measurements Achieved during Sandbox Experiments

**DOI:** 10.3390/s20020570

**Published:** 2020-01-20

**Authors:** Nataline Simon, Olivier Bour, Nicolas Lavenant, Gilles Porel, Benoît Nauleau, Behzad Pouladi, Laurent Longuevergne

**Affiliations:** 1 Univ Rennes, CNRS, Géosciences Rennes, UMR 6118, 35000 Rennes, France; nicolas.lavenant@univ-rennes1.fr (N.L.); behzad.pouladi@univ-rennes1.fr (B.P.); laurent.longuevergne@univ-rennes1.fr (L.L.); 2 IC2MP, Univ. Poitiers, CNRS UMR 7285, 86022 Poitiers, France; gilles.porel@univ-poitiers.fr (G.P.); benoit.nauleau@univ-poitiers.fr (B.N.)

**Keywords:** fiber-optic distributed temperature sensing, spatial resolution, laboratory experiment, active-DTS method

## Abstract

For many environmental applications, the interpretation of fiber-optic Raman distributed temperature sensing (FO-DTS) measurements is strongly dependent on the spatial resolution of measurements, especially when the objective is to detect temperature variations over small scales. Here, we propose to compare three different and complementary methods to estimate, in practice, the “effective” spatial resolution of DTS measurements: The classical “90% step change” method, the correlation length estimated from experimental semivariograms, and the derivative method. The three methods were applied using FO-DTS measurements achieved during sandbox experiments using two DTS units having different spatial resolutions. Results show that the value of the spatial resolution estimated using a step change depends on both the effective spatial resolution of the DTS unit and on heat conduction induced by the high thermal conductivity of the cable. The correlation length method provides an estimate much closer to the value provided by the manufacturers, representative of the effective spatial resolutions along cable sections where temperature gradients are small or negligible. Thirdly, the application of the derivative method allows for verifying the representativeness of DTS measurements all along the cable, by localizing sections where measurements are representative of the effective temperature. We finally show that DTS measurements could be validated in sandbox experiments, when using devices with finer spatial resolution.

## 1. Introduction

Initiated in the 1980s, the use of fiber-optic distributed temperature sensing (FO-DTS) has been considerably improved, and this technology is nowadays widely applied in a large range of applications in various scientific and industrial disciplines, such as the oil and gas industry, leakage and fire detection, structure health monitoring, civil engineering, etc. [1,2]. Over the last decade, the ability of this tool in hydrology has been widely demonstrated [3], especially for soil moisture determination [4,5], surface waters monitoring [6,7], hydrogeological applications [8], and at the groundwater–surface water interface [9,10,11]. In a commentary, Shanafield et al. highlighted the increase of environmental application of DTS and identified around 500 peer-reviewed articles published in hydrologic applications in seven years [12].

The emergence of DTS applications has been enabled by the improvement of the method and the increase of the quality of collected data. Thus, works have been dedicated to metrological characterization and specification of DTS devices (principle and physic of the measurement, technology limitations) [6,13,14,15,16,17], improvements in calibration methods [18,19], and in instrumentation deployment (DTS unit and cable selection, field installation) [8,13,15]. In this context, Tyler et al. [13], Selker et al. [15], and Failleau et al. [17] demonstrated the importance of considering the spatial resolution of DTS systems. Spatial resolution can be defined as “the spatial integration scale over which a single value of temperature is reported” [13]. In fact, each collected data corresponds to the average of temperature along a certain length of fiber, meaning that collected data at sample spacing are not truly independent of their adjacent samples. Thus, Selker et al. [15] suggested that the temperature reported at each sample interval is an average weighed by a Gaussian function centered at the sampling interval, with a standard deviation equivalent to the spatial resolution. It implies the differentiation between the spatial resolution (also defined as the minimum distance to detect a step change in temperature) and the sampling interval (distance between two successive measurement points). This distinction is required to define the ability of the device to detect punctual change in temperature and to fully understand the meaning of collected data [13,15,17]. 

Broadly speaking, the spatial resolution is, in general, larger than the sampling interval. According to the Nyquist theorem, the value of the spatial resolution can be considered at least twice the value of the sampling interval [15]. The spatial resolution of a Raman DTS system depends on dispersion of light along the fiber, but also on technical characteristics such as the frequency of the laser, the signal generating, or the limitations of the detector [13]. Thus, DTS instrument manufacturers define the specifications of each DTS device, including the minimum sampling interval and the spatial resolution. For Raman spectra DTS, the highest performing devices can provide a 0.125 m sampling interval with a 0.29 m spatial resolution, whereas some devices provide a 2 m sampling interval with a 4 m spatial resolution [12,15].

In environmental sciences, and especially in geosciences, FO-DTS technology has been widely applied considering its ability to monitor spatial and temporal temperature changes with a high-accuracy [12,18]. The value of the spatial resolution controls partly the ability of the user to detect local temperature variations, and the choice of the DTS device depends on the application and on the scale of temperature contrast [17]. Note that, in any case, there is a trade-off since a better spatial resolution may lead to a lower temperature resolution. In many studies, the manufacturers’ specifications are mentioned but the spatial resolution of DTS unit is often not considered in the interpretation of the data. In such case, results should be interpreted cautiously. When it has been taken into account, it has been shown that the limit of identification of hydrogeological structures, such as individual fractures or layer of sediments, is directly dependent on the spatial resolution of the DTS unit [8,20,21].

The spatial resolution can be estimated experimentally by applying the “90% step change” method [13]. It consists of observing the ability of the DTS unit to measure a sharp step change in temperature occurring along the cable and defining the distance necessary to measure 90% of the step change [13,22,23]. As pointed out by Tyler et al. [13], the spatial resolution, observed and measured during field applications, depends both on the unit and on the fiber-optic cable used. Thus, significant differences may be observed between the effective spatial resolution, estimated during field applications, and the spatial resolution provided by the manufacturers. This difference may be due to laboratory conditions (cable, calibration baths) used when estimating the spatial resolution, but also to testing methodology. DTS manufacturers widely apply the “10–90 method” to determine the spatial resolution, thus considering the length required to monitor 80% of the step change. Estimated values of spatial resolution in the field could be up to 10 times larger than manufacturer specifications [13,15,17], meaning that the effective spatial resolution should be estimated for each fiber–instrument combination and each individual application [13].

This difference between manufacturers’ specifications and effective spatial resolution raises the question of the meaning and the interpretation of collected data, especially during laboratory tests, where spatial resolution can be close to the size of the experiment. Only a few studies have attempted laboratory tests based on Raman spectra FO-DTS [24,25]. These studies rely only on the value of the spatial resolution provided by manufacturers, while such assumption can directly affect the interpretation of collected data. A striking example is the study of Mamer and Lowry, who conducted laboratory experiments on the quantification of discharge using paired fiber-optic cables [24]. They concluded that the underestimation of localized discharge zones could directly be attributed to the spatial resolution of the DTS unit, actually larger than the one specified by the manufacturer. 

In this context, this study raises the question of the feasibility and the validation of FO-DTS measurements conducted in laboratory tests. We propose and compare here different methods that allow for estimating the practical or effective spatial resolution of DTS systems. It is first estimated using the classical “90% step change” method, allowing a comparison with manufacturers’ specifications, although the laboratory conditions are not rigorously similar to the ones provided by manufacturers. To disregard the potential effect of the fiber-optic cable on the estimate, we also propose to evaluate the effective spatial resolution by using two other complementary methods. Thus, correlation lengths of measurements within calibration baths are estimated through the analysis of semivariograms. Finally, a simple method based on the derivative of temperature with respect to distance measurements is presented. Besides estimating the effective spatial resolution of measurements, this approach allows for validating the representativeness of DTS measurements all along the cable. Laboratory tests were conducted using two DTS devices connected to a single fiber-optic cable, providing simultaneous temperature measurements with two different spatial resolutions. We finally discuss the capability of conducting measurements in the laboratory tests and the application of DTS technology in various environments.

## 2. Materials and Methods

### 2.1. The “90% Step Change” Method

The spatial resolution can be defined as the minimum distance of cable required to fully detect a localized temperature step occurring along the cable. It depends on technical specifications of the DTS unit (optical pulse width and electronic bandwidth) and on the dispersion of the light along the glass fiber. In practice, manufacturers generally follow recommendations describing test procedures to estimate the spatial resolution. Detailed information may be found in the SEAFOM’s document (Subsea Fiber-Optic Monitoring Group, Epsom, UK; SEAFOMMSP-01) [23] and the publication of the COST (European Action for Cooperation in Science and Technology; COST-299) [22], that are reference documents considering the specifications and characterizations of DTS measurements.

Test procedures consist, generally, in estimating the spatial resolution as the minimum distance that allows the measurement of 90% of an abrupt step change. The procedure can consist of immersing increasing lengths of cable inside a calibration bath and estimating the length required to detect at least 90% of the temperature step [17,22,23]. Similarly, Tyler et al. showed that this method could be applied directly by considering just one single test setup, by estimating the length over which the DTS unit shows 90% of a step change in temperature. It requires generating a sharp step change in temperature along the cable, surrounded by long enough sections of cable maintained at a constant temperature [13]. Note; however, that some publications suggest applying the step change method by considering the length required to monitor from the 10% to the 90% level of the step change in temperature (“10–90 method”) [1,15]. Since this method is based on the observation of 80% of the step change, the result will suggest a finer spatial resolution compared to the 90% step change method. The “10–90 method” is also widely applied by DTS manufacturers to determine the spatial resolution [15]. Note that this method is presented as an additional method to estimate the spatial resolution in the SEAFOM’s document. Nevertheless, it specifies that the measured “10%–90%” value should be multiplied by 1.31 to get an estimation of the spatial resolution estimated through the 90% step change method [23]. The latter point is generally neglected. This lack of standardization makes comparison between units and manufacturers difficult. In the following, we will apply only the “90% step change” method, considering the spatial resolution as the length required to monitor from 5% to 95% of the temperature step change. In the field, this method may be applied using the inlet and the outlet of calibration baths that ensure the presence of a sharp step change in temperature along the cable [13]. 

However, it may be difficult to compare the estimates of spatial resolution in the field with the DTS units’ specifications provided by the manufacturers. DTS units’ specifications are defined in ideal laboratory conditions, based on abrupt transitions between chambers where measurement conditions and temperature are perfectly controlled. In field conditions, cold or warm baths are generally used but the generated step change in temperature is not as abrupt as during ideal laboratory conditions. Then, Tyler et al. showed that the quality and characteristics of fiber-optic cable could affect the spatial resolution of DTS systems [13]. One of the main limitations of this method is that the step change induces lateral heat conduction along the cable according to the thermal conductivity of the cable. Manufacturers generally use bare fiber (i.e., glass with polymer coating but without cable protection), which allows for neglecting the effect of thermal conduction along the cable, especially for an abrupt temperature step change. The monitored step change of temperature along the cable depends; thus, on the spatial resolution of the DTS unit but also on the heat conduction along the cable that may partly blur the estimation of the spatial resolution. The application of the “90% step change” method can; thus, lead to coarser effective values of the spatial resolution, depending on the thermal properties of the cable. Here, we propose, in the following, the use of complementary methods in order to characterize the effective spatial resolution and the representativeness of DTS measurements all along the cable.

### 2.2. Estimation of the Effective Spatial Resolution

First, the effective spatial resolution was estimated with the classical “90% step change method” described by Tyler et al. [13] using the inlet and the outlet of calibration baths. Note that the SEAFOM’s report recommends applying an interpolation method before estimating the step change, which was not applied here. Since sufficient data points were available to describe the step change, an interpolation would not significantly change the results obtained.

We also propose to calculate the semi-variance of measurements that allows for defining the spatial dependence between successive observations. The analysis of the semivariogram provides information about the correlation length between measurements from each DTS unit. Semi-variance can be calculated using long enough sections of cable deployed in homogeneous and constant temperature areas, thus not necessarily warm or cold calibration baths. Contrary to the “90% step change” method, the heat conduction along the cable can be neglected in homogeneous sections where no temperature gradients or variations occurs. The correlation length should; therefore, provide an estimate of the spatial resolution much closer to the one provided by the manufacturers, independently of physical processes, such as heat conduction, along the FO cable. The semivariogram γ(h) is given by [26,27]:

(1) $$\gamma \left(h\right)=\;\frac{1}{2N\left(h\right)}\sum_{i=1}^{N\left(h\right)}\left(z\left(x_{i}\right)-z\left(x_{i}+h\right)\right)²,$$

where z(xi) and z(xi + h) are temperature measurements at locations (xi) and (xi + h), h being the lag distance (i.e., the distance between two pairs of data points located at i and j) and N(h) the number of data pairs separated by h. The spatial dependence of data is then estimated by determining the parameters of semivariograms, especially the range, which is an estimate of the maximum correlation length between two points at separation distance h. At distances smaller than the range, a spatial correlation exists between data points, while measurements are supposed to be uncorrelated at greater distance.

Semivariograms can be constructed with data collected in calibration baths and can be interpreted by fitting a Gaussian model to experimental data:

(2) $$\gamma \left(h\right)={\;C}_{0}+{\;C}_{1}\left[1-e^{\left(\frac{-3h^{2}}{\alpha^{2}}\right)}\right],$$

where C_0_ is the nugget, C_1_ the sill, and α the range of influence. The nugget effect C_0_ can be interpreted as random noise, as short-scale variability, or as the measurement error [28]. The semivariograms were interpreted by fitting a Gaussian model (Equation (2)) on experimental data to estimate the nugget effect C_0_ and the range of influence α. 

In theory, the estimation of the effective spatial resolution using the “90% step change” method and the estimation of the correlation length of measurements should provide two complementary estimates of the effective spatial resolution. Nevertheless, in the field or during a lab experiment, the high variability of temperature that may occur along the cable raises the issue of the meaning and the representativeness of measurements. In such case, it may be difficult to know which of these values should be considered along the cable. In areas where no temperature changes are observed, the correlation length may be better suited, while in areas characterized by large temperature step changes, the effective spatial resolution estimated from the step change method should be more appropriate. 

To go further, we propose to evaluate the spatial temperature variations along the cable using the “derivative method”. It consists of calculating the derivative of the temperature with respect to distance measurements all along the measurement length:

(3) $$\frac{\mathrm{dT}}{\mathrm{dx}}=T_{n}^{\prime }=\frac{T_{n+1}-{\;T}_{n}}{x_{n+1}-x_{n}},$$

where T_n+1_ and T_n_ are temperature measurements at locations x_n+1_ and x_n_. The difference (x_n+1_ − x_n_) represents the sampling interval. 

Theoretically, when temperature remains stable over a sufficient distance or when temperature changes occur at a scale larger than the effective spatial resolution, temperature variations along the cable are particularly smooth or negligible. This implies that the gradient in temperature between successive measurement points is low, meaning that the derivative of temperature should tend to zero. Conversely, when abrupt temperature changes occur along the cable due to thermal anomalies, local peaks, step changes, etc., the DTS device may not be capable of fully measuring the temperature signal. In this case, the gradient or derivative of temperature should digress from zero. To summarize, the spatial derivative of measurements, through the estimation of the spatial variability of measurements along the fiber-optic cable, should provide a good estimation of the representativeness of temperature measurements with respect to the effective spatial resolution.

### 2.3. Modelling FO-DTS Measurements

**Figure 1:**
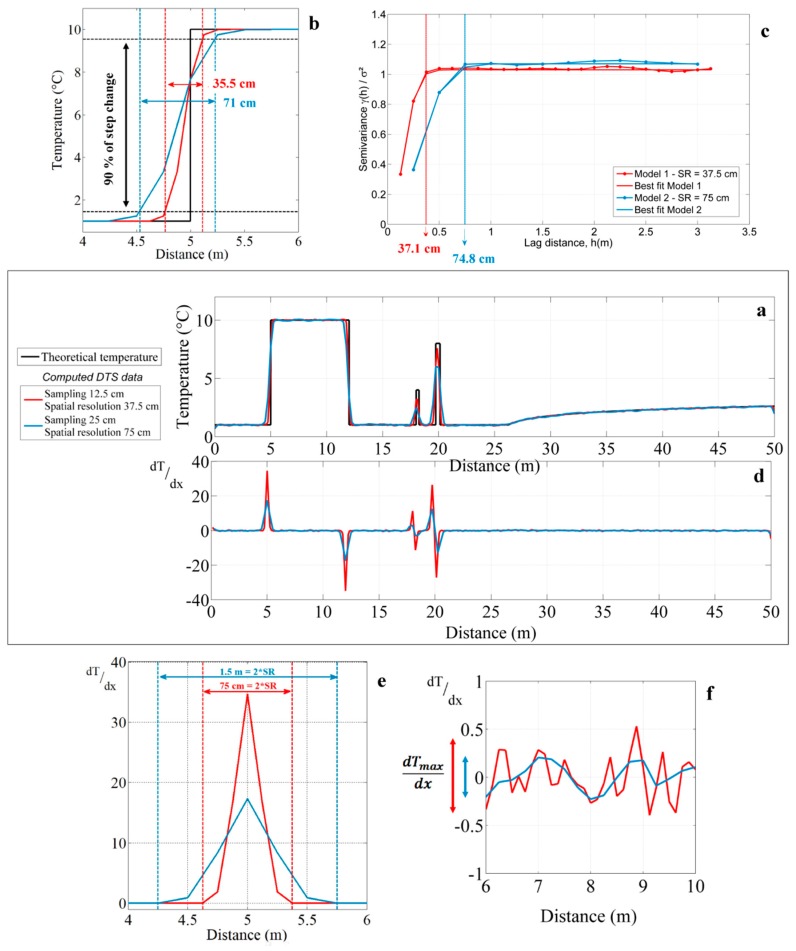
(**a**) Numerical modelling highlighting the impact of spatial resolution on Distributed Temperature Sending (DTS) measurements. The red and blue lines correspond to modelled temperature profiles and the black line to the synthetic temperature profile. (**b**) Estimation of the spatial resolution using the “90% step change” method. (**c**) Semivariograms computed from modelled temperature profiles using data between 6 and 11 m (step change). The spatial resolution can be estimated by fitting a Gaussian model on semivariograms (solid lines) and estimating the correlation length (range). (**d**) Derivative of the temperature estimated with respect to distance along the cable for both modelled temperature profiles. (**e**) Estimation of the spatial resolution using the derivative method applied on sections where a sharp step change occurs along the temperature profile. (**f**) Derivative calculations in constant and homogeneous sections of temperature.

To illustrate the different approaches used to estimate the effective spatial resolution of DTS measurements, an analytical model reproducing DTS measurements was developed. An arbitrary temperature profile was first postulated (Figure 1a, black line) for reproducing DTS measurements according to sampling interval, spatial resolution, and noise measurement (Figure 1a, red and blue lines). We assumed the different measurement points as the weighted sum of the unit Gaussian distribution, with the spatial resolution as standard deviation multiplied by the actual temperatures, as suggested by Selker et al. [15] and applied by Maldaner et al. [21] and Bense et al. [8]. DTS data were modelled considering either a 12.5 cm sampling interval and a 37.5 cm spatial resolution (red line), or a 25 cm sampling interval and a 75 cm spatial resolution (blue line). These values were chosen arbitrarily in accordance was added to the data in order to reproduce noise measurements occurring during DTS data acquisition. This 50 m synthetic temperature profiles allowed for simulating the effect of an abrupt step change from 7 to 12 m, of localized thermal anomalies at 18 and 20 m, and of a smooth increase of temperature from 27 to 50 m. 

As shown in Figure 1a, simulated temperature profiles (red and blue lines), reproducing DTS measurements, are in perfect agreement with the synthetic temperature profile (black line) when temperature variations are smooth (from 27 to 50 m) or when temperature is constant over a sufficient length. Here, this means that whatever the spatial resolution assumed, DTS measurements are representative of the effective temperature along these sections. On the opposite, DTS measurements are not capable of efficiently monitoring step changes in temperature occurring right at the step change, respectively located at 5 and 12 m along the temperature profile (Figure 1a), as highlighted in Figure 1b. As expected, the length required to monitor the step change is directly dependent on the spatial resolution of DTS units. Likewise, DTS measurements are not representative of the effective temperature and temperature changes are widely underestimated when local thermal anomalies occur along the profiles (peaks in temperature at 18 and 20 m, as shown in Figure 1a).

The different approaches used to estimate the spatial resolution of DTS measurements can be applied using the different modelled temperature profiles. As shown in Figure 1b, when considering the “90% step change” method, the length required to monitor 90% of the step change is 35.5 cm for the first model (red line) and 71 cm for the second one (blue one), which are in very good agreement with the values of spatial resolutions assumed for the simulations (37.5 and 75 cm, respectively). When applied at 12 m, at the outlet of the temperature step, similar results are obtained. As shown in Figure 1c, calculating semivariances of simulated data on a cable section where temperature is roughly constant, for instance from 6 to 11 m, provides also very good estimates for the correlation lengths that are equal to 37.1 cm for the first model (red line) and 74.8 cm for the second one (blue line). This demonstrates that estimating the correlation length provides a very good estimation of the spatial resolution. Theoretically, the spatial resolution and the correlation length are; thus, equivalent and both the “90% step change” method and the correlation length estimates could be applied interchangeably. 

The derivative method can then be applied to evaluate the representativeness of temperature measurements all along the cable. Figure 1d shows the evolution of the derivative of the temperature with respect to distance, calculated for both modelled temperature profiles. The derivative is close to zero when temperature variations are smooth (from 27 to 50 m) or when temperature is constant over a certain length (i.e., along the baseline of temperature). By focusing on these sections, as shown in Figure 1f, it can be highlighted that the derivative fluctuates actually within a narrow range close to zero. This range corresponds to the maximum temperature difference occurring between two successive measurement points in homogeneous temperature sections, and is around 0.85 °C/m for the first model (red line) and 0.4 °C/m for the second one (blue line). By multiplying this value by the sampling interval, it allows for evaluating the noise measurement at 0.1 °C for both models, which is fully consistent with the input model.

However, derivative reaches ±35 °C/m for the first model (red line) and ±27 °C/m for the second one (blue line) right at step changes at 5 and 12 m along the temperature profile. As shown in Figure 1e, when a step change in temperature occurs along the cable, the distance over which the derivative varies from zero is twice the length of the spatial resolution. Thus, this suggests that the spatial resolution can also be estimated directly using the derivative method at a step change surrounded by long enough sections of cable maintained at a constant temperature. In the case of local thermal anomalies at 18 and 20 m, as shown in Figure 1d, the estimation of the spatial resolution is not possible due to a too limited number of data points that describes such variations. However, the very large variations of the derivative around these anomalies reveal cable sections where temperature measurements are not representative of the effective or “true” temperature. In conclusion, this method allows for going further by evaluating the robustness of measurements all along the cable, simply by estimating the derivative of temperature measurements along the cable.

### 2.4. Sandbox Experiments

#### 2.4.1. Objectives of the DTS Experiments

The experimentation consists in conducting a succession of laboratory active-DTS experiments. The active-DTS method was recently developed and consists of continuously monitoring the change in temperature along a heated cable. The elevation in temperature depends on sediments thermal properties but also on groundwater flow velocities that dissipate heat through advection [20,25,29,30,31]. In the present case, the experiment was based on the electrical heating of the cable through its steel frame injection, meaning that the fiber-optic cable was both the heating source and the measurement point. The aim of these laboratory tests is to quantify the effect of flow rate conditions on the thermal response measured along the cable for validating some analytical solutions. Another objective relies on the understanding of the effect of the angle between the flow direction and the fiber-optic cable on measurements. This requires a specific and relevant installation of the fiber-optic cable in the sandbox with accurate characterization of the spatial resolution of measurements during the experiment. Indeed, due to the limited size of the sandbox experiment compared to spatial resolution of measurements, this may require a point-by-point analysis of measurements. Here, we present the comparison of different methods to estimate the spatial resolution and the interpretation of active-DTS experiments is beyond the scope of the study. In the following, we present briefly the experimental setup that allowed the acquisition of DTS measurements. 

#### 2.4.2. Experimental Setup

**Figure 2:**
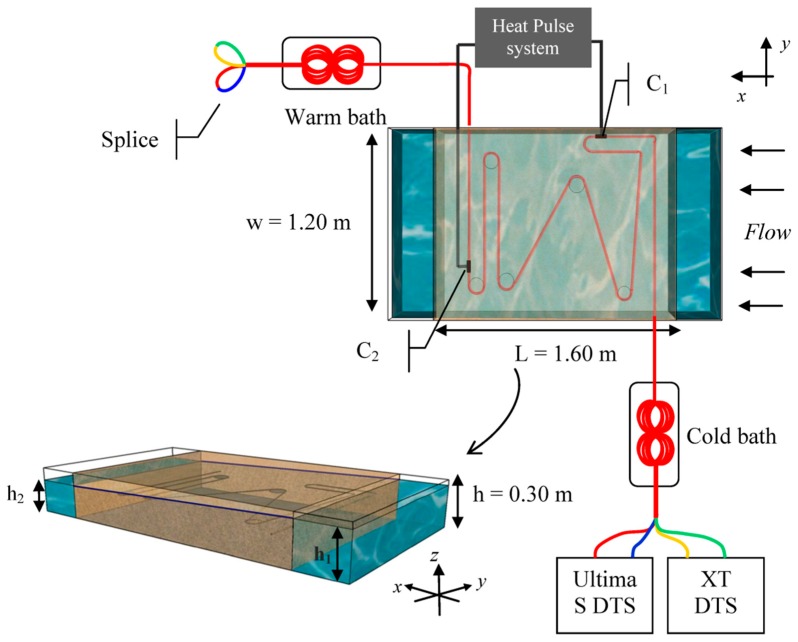
Experimental setup for Distributed Temperature Sending (DTS) measurements showing the dimensions of the sandbox and the deployment of fiber-optic cable (top view (x,y plane) and side view (x,y,z plane)). Each paired fiber was respectively connected to a Silixa Ultima S DTS unit and to a Silixa XT-DTS unit. The marks C_1_ and C_2_ represent connection points between fiber-optic cable and electrical cables allowing the injection of electricity into the steel frame of the cable.

The experimental setup is illustrated in Figure 2. The sandbox was a 0.576 m^3^ PVC tank open at the top (1.6 m length, 1.2 m width, and 0.3 m height) and filled with sand. Inlet and outlet of water were controlled by water level in both water reservoirs on either side of the sandbox. Both sides were consistently perforated allowing an equal distribution of incoming and out coming water over the width and the height of the sandbox. The water circuit was a closed loop. First, a pumping system supplied water from a separate larger tank toward a degasification system. This process aims to limit the formation of air bubbles into the sand. Then, the degassed water was sent toward the inlet reservoir. The water level (h_1_) in this reservoir was controlled by an overflow outlet, whose height could be manually adjusted. Next, water flowed through the sand up to the outlet reservoir, where water level was also controlled by an overflow outlet. Finally, excess of water was sent back towards the separate tank before being re-injected into the system.

**Figure 3:**
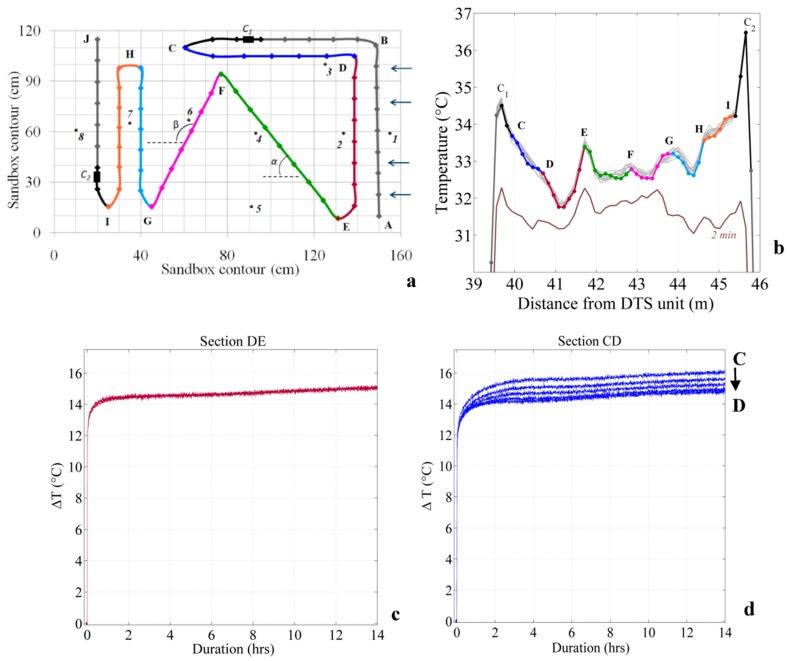
(**a**) Schematic top view of the deployment of the fiber-optic cable in the sandbox. Temperature is recorded from point A to point J. The different cable sections were highlighted by different colors. Dots along the cable represent the localization of measurement points recorded with the Ultima S DTS (Distributed Temperature Sending) unit. Electrical/fiber-optic cable connections are represented by black rectangles (C_1_ and C_2_). Black numbered dots represent PT100 probes installed in the sandbox. (**b**) Temperature profiles recorded with the Ultima S DTS unit along the heated section during the first heating period. The brown line corresponds to the temperature profile recorded after the first two minutes of heat injection. All other lines (gray and colored lines) highlight the temporal variability of temperature profiles after temperature stabilization (more than 4 h of heating) and correspond to temperature profiles recorded one after the other at sampling interval (20 s) during 4 min. The different cable sections were highlighted on the temperature profile with the same colors as (a). (**c**) Evolution of ∆T over time during the third heating period along the section DE. The three external points were removed here. (**d**) Evolution of ∆T over time during the third heating period along the section CD.

The first 10 cm of the tank was filled manually with 0.4–1.3 mm diameter sand to get a flat and smooth surface of sand. Then, 9 m of fiber cable was deployed on the top of this surface so that the entire cable lies at the same depth. As shown in Figure 2, the cable was installed in the sandbox so that the angle between the flow and the fiber-optic cable varied in space. The aim was to estimate the effect of this angle on temperature measurement. A 7 m section of heated cable was electrically isolated and connected to electrical cable (connections C_1_ and C_2_) allowing the injection of electricity by connecting to a power controller. The cable was maintained in shape within the sandbox through PVC discs (diameter 10 cm) installed at each change in cable direction, at the end of each cable section. No PVC disc was required at the intersection between sections CD and DE (see Figure 3a). Lastly, the tank was fully filled with the same sand. Given the uniform setting of the sand, flow was considered homogeneous within the sandbox. The sand was saturated in water and flow rate was maintained as constant and maximum as possible during 24 h to ensure flow homogenization within the box. From now on, this stabilization period will be referred as “the start of experiment”. During the experiment, the water level (h_1_) was systematically kept 5 cm below the top of the sand to prevent water from flowing over sediments. Flow rates were measured independently and manually at the outlet of the sandbox, allowing for the calculation of the hydraulic conductivity using Darcy’s law under unconfined conditions and estimated at 3.1 × 10^−3^ ± 8 × 10^−4^ m/s.

#### 2.4.3. Data Acquisition

The fiber-optic cable used is a 3.8 mm-diameter fiber-optic cable (BruSens cable; reference LLK-BSTE 85 °C) containing 4 multimode 50/125-µm fibers. The cable is armored with a stainless steel loose tube surrounded by stainless strength members and a polyamide outer sheath. Through this armored protection, the thermal conductivity of the cable is particularly high, ensuring a fast thermal response and simplifying the conduction of heat experiments within the cable. However, it also permits conductive heat transfer along the cable. As shown in Figure 2, fibers were paired and splices were made at the end of the cable, allowing measurements in double-ended configuration [19]. Thus, temperature can be simultaneously monitored all along the cable by connecting each paired-fiber to a FO-DTS control unit. This configuration is ideal to compare DTS units’ performances, since temperature is monitored at the same time in the same cable. The first paired-fiber was connected to a Silixa Ultima S DTS unit (Silixa Ltd., Elstree, UK), reported temperature every 12.5 cm with a 33 cm spatial resolution. The second one was connected to a Silixa XT-DTS unit (Silixa Ltd., Elstree, UK), reported temperature every 25 cm with a 54 cm spatial resolution. Note that these values are the ones provided by the manufacturer specifically defined for these two units and obtained applying the 10–90 method” (i.e., the length required to monitor 80% of a step change). Therefore, the sampling interval of the XT-DTS unit is twice larger than the Ultima S unit, meaning that the number of samples is divided by two. DTS units were configured to collect data at a 20 s sampling interval (10 s per channel). As shown in Figure 3a, the cable was installed in the sandbox so that the angle between the flow and the fiber-optic cable varied in space. Thus, cable sections called DE, HG, and HI are perpendicular to water flow, whereas cable section CD is parallel to water flow. Sections EF and FG lie, respectively, at a 60° angle and at a 110° angle to water flow. Cable sections were installed long enough to insure recording several measurement points before any change of direction. Thus, the cable section lengths in the sandbox varied between 0.9 and 1 m, providing 3 or 4 measurements points per section for the XT DTS unit and 7 or 8 measurements points per section for the Ultima S DTS unit.

To calibrate DTS units, a 20 m length of cable section was placed in a cold calibration (box filled with wetted ice) bath and a 20 m length in a warm calibration bath (equipped with a heating resistor). In each bath, a PT100 probe (0.1 °C) and an RBR SoloT probe (0.002 °C accuracy) recorded the temperature and air pumps were set to homogenize temperature. Temperature was monitored independently at several places along the experimental setup as well. Thus, RBR SoloT probes were installed within a 5 m loop of fiber set between the cold bath and the sandbox, allowing the evaluation of DTS data quality. Others RBR temperature sensors were installed in the inlet and outlet reservoirs and collected data were compared with FO-DTS data. Lastly, 8 PT100 probes were set directly inside the sandbox to monitor temperature evolution at different distances from the fiber-optic cable, as shown in Figure 3a. Relative uncertainty of temperature measurements, also called temperature resolution, can be estimated at 0.03 °C in calibration baths. Absolute uncertainty of measurement can be estimated at 0.15 °C by comparing DTS measurements and RBR soloT probes in calibration baths. Such uncertainty highlights the advantage of the double-ended measurement [19]. The robustness of the configuration and the calibration was also confirmed since attenuation occurring along the cable was corrected and temperature measurements are similar before and after the splice. The consistency of measurements between the two devices was checked by calculating the difference of measurements, monitored over time in calibration baths, external sections (air temperature), and in the sandbox outside of heating periods. The values of standard deviation of measurements, calculated for each device, were also compared. Thus, the difference of standard deviations between the two units is very low (<0.02 °C), meaning that both devices record the same variability of temperature over time. Then, the average difference of temperature measured all along the cable between both devices is around 0.06 °C, confirming that temperature measurements in the sandbox, outside of heating periods, are quite similar with both devices.

#### 2.4.4. Heat Tracer Experiment

The experimentation consists of conducting a succession of laboratory active-DTS experiments under different flow rate conditions. After changing the flow rate and before starting active-DTS experiments, a more or less long interval time was necessary to reach hydraulic steady state, depending on flow conditions. After that, the cable was energized continuously during a few hours (between 6 and 8 h) using a Silixa’s Heat Pulse Control System. The total electrical power injected was calculated in order to deliver a power intensity of 20 W/m along the heated section. The heat pulse system is controlled by CCI Control Panel software v.2.7.9 that records continuously the power supply specifications. It confirmed that the power controller supplied a quasi-constant and homogeneous power over time. Lastly, the recovery period was monitored all night long after turning off the power controller. This way, the full return to the initial state was ensured. It should be noted that the inflow temperature changed over time by a few degrees around the mean temperature of 20 °C (±2 °C). This change can be explained by air temperature changes inside the experimental room, by the potential warming of water in the tank induced by the pump and by the global heating up of the water in the tank induced by heating periods (closed loop water circulation system). Since these variations had an impact on temperature changes during heating and recovery periods, data were post-processed by applying a filter based on the propagation and the attenuation of temperature changes inside the sandbox using classical heat transport equations [32,33].

While the temperature is expected to be broadly uniform in the sandbox, the application of the active-DTS experiment in the sandbox generates artificial temperature contrasts, as shown in Figure 3b. First, the temperature measured from the cable section DE to the cable section HI tended to increase. In other words, a gradient of temperature between the inlet and the outlet of the sandbox is created because of heat transport and heat accumulation in the sand. Thus, the increase of temperature recorded along the section HI depends on the local increase of temperature induced by heat injection along the section, but also on the transport of heat produced upstream in the sandbox. On the opposite, the temperature decreases steadily along the cable section CD, since temperature is recorded from downstream to upstream along this section. Secondly, as shown in Figure 3b, higher temperatures are recorded at the two ends of each sections (peaks at points E, F, G, and H). This is due to (i) the proximity of different heated cable sections—different heating sources—next to each other in these areas, but also to (ii) the presence of PVC discs installed at the ends of linear cable sections to hold the cable in the sandbox. Lastly, as highlighted in Figure 3b, the temperature profile shows sudden and sharp temperature peaks at the beginning and at the end of the heated section (C_1_ and C_2_). This is the result of electrical/fiber-optic cables connections, sealed by epoxy resin, that induce local thermal properties changes during heating periods.

To summarize, the temperature profile is highly variable along the cable into the sandbox during active-DTS experiments. However, the shape of this profile is quite similar during the full duration of the heating period, starting from the first instants of heat injection, as highlighted in Figure 3b. Thus, despite the apparent spatial variability, the evolution of the ∆T over time is consistent and not as variable as expected, as shown in Figure 3c,d. Thus, while the thermal response is similar at all measurement points along the section DE (see Figure 3c), ∆T increases from upstream to downstream (from D to C) because of heat transport along the cable according to the flow rate (see Figure 3d). 

## 3. Results

### 3.1. Effect of Spatial Resolution on Temperature Profiles

**Figure 4:**
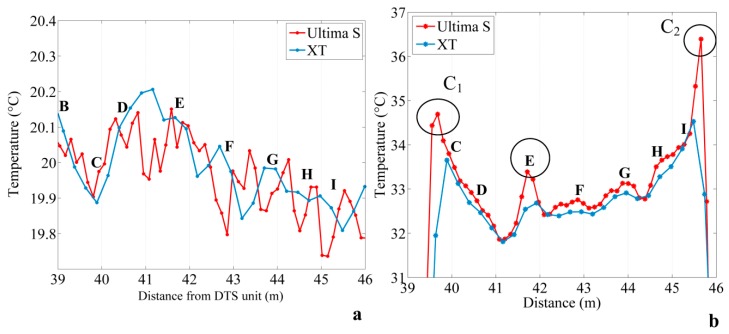
(**a**) Example of temperature profiles measured with the XT DTS (Distributed Temperature Sending) unit and the Ultima DTS unit in the sandbox at a non-heated time (**b**) Example of temperature profiles measured with the XT DTS and the Ultima DTS unit in the sandbox at a heated time, after temperature stabilization.

Figure 4a shows an example of temperature profiles obtained with both DTS units in the sandbox at a non-heated time. Results are similar with the two devices within noise measurement. The temperature decreases along the section BC, then increases along the section CD, and finally decreases from section DE to section IJ. In other words, at this time, the temperature decreases from upstream to downstream in the sandbox. Outside of heating periods, temperature changes depend on temperature variations in the inlet and flow rate that controls the residence time of water inside the sandbox. Residence times varied between 1 and 3 h depending on the flow rate. During these periods, both units record the same trend. The mean temperature and the standard deviation are also equivalent independently of DTS performances, with a mean difference of 0.06 °C. However, data collected using the Ultima S DTS unit seem a bit noisier because of the finer sampling interval.

In contrast, the difference of temperature is higher between the two units during heating periods, with a mean difference of 0.42 °C, as shown in Figure 4b. Broadly speaking, both units record the same trend of temperature, namely a decrease of temperature along the section CD, then an increase from upstream to downstream in the sandbox, as described in the Section 2.4.3. However, significant differences are observed between the two temperature profiles. The Ultima S DTS unit records higher temperatures than the XT DTS unit and allows for the more efficient detection of local peaks of temperature depicted by open circles on Figure 4b. Temperature measurements recorded with the XT DTS unit are on average 25% less variable than measurements recorded with the Ultima DTS unit. Since the consistency of temperature measurements between the two devices was verified in the warm calibration bath, we assume that this difference is directly due to the DTS units’ performances. Thus, through a finer sampling interval and spatial resolution, the Ultima S DTS unit more efficiently highlights local contrasts of temperature. It should be noted that local increases in temperature are also visible with the XT DTS unit, since the variability of temperature measurements during heating periods is up to three times as much as during recovery periods. These contrasts are; however, underestimated and slightly shifted in space due to a coarser spatial resolution. Elsewhere, both DTS performances are quite similar, illustrating that the sampling interval and the spatial resolution do not affect the ability of temperature measurement, while temperature variations are smooth along the cable or while temperature changes occur over sufficiently long sections of cable.

### 3.2. Estimation of the Effective Spatial Resolution

#### 3.2.1. Using the “90% Step Change” Method

**Figure 5:**
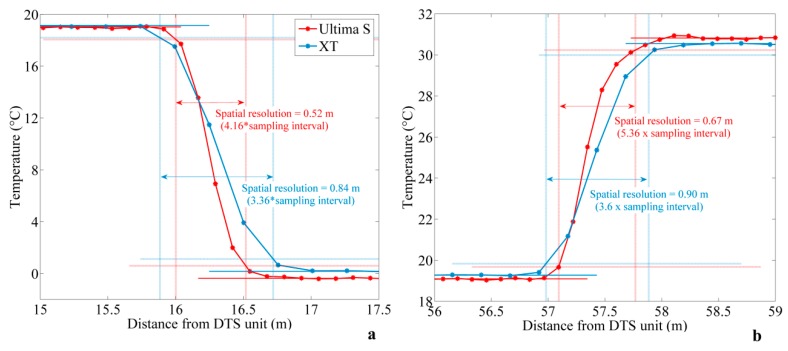
Estimation of the spatial resolution using the “90% step change” method for both DTS (Distributed Temperature Sending) devices, collected at the sampling interval (Ultima S DTS: Day 1—02:02:14 p.m.; XT DTS: Day 1—02:02:01 p.m.) (**a**) at the inlet of the cold bath and (**b**) at the inlet of the warm bath.

Figure 5a,b shows examples of the estimation of spatial resolution for each DTS unit using the “90% step change” method introduced by Tyler et al. [13], applied at the inlet of the cold bath and the inlet of the warm bath, respectively. The method was also applied at the outlet of the baths and at different times but, given the similarity in results, these applications are not presented here. Unsurprisingly, the distance required to detect a step change is systematically lower for the Ultima S DTS unit, meaning that its spatial resolution is finer than the spatial resolution of the XT DTS. This result is in agreement with the DTS units’ specifications provided by the manufacturer. 

However, the effective spatial resolution estimated from our data points varies between 52 and 67 cm for the Ultima S DTS and between 84 and 90 cm for the XT DTS unit. In other words, the effective spatial resolutions for both DTS units are between one and a half and two times larger than the one provided by the manufacturer, defined as equal to 33 cm for the Ultima S DTS unit and 54 cm for the XT DTS unit using the “10–90 method”. These results suggest that the Ultima DTS unit requires between four and six points of measurements (at 12.5 cm interval sampling) to detect a step change, whereas the XT DTS unit requires between three and fouru points of measurements (at 25 cm interval sampling) to detect the same change. Below these values, thermal anomalies may not be fully detected and can be underestimated, in particular if the contrast in temperature is particularly localized.

Differences observed between the two calibration baths (see Figure 5) are representative of the variability observed when estimating the spatial resolution at sampling interval. Thus, an automatic script allowed for applying and repeating the method for different sampling intervals. The integration of data over greater time (1 then 5 h integration time) led to reduce the variability of estimated spatial resolution, and consequently improve the estimation of the effective spatial resolution. 

#### 3.2.2. Using the Correlation Length

**Figure 6:**
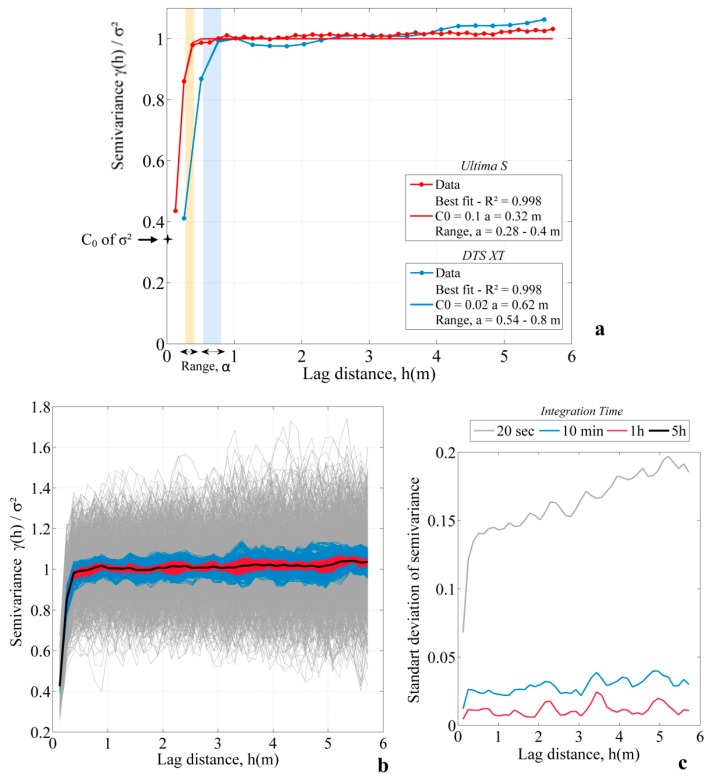
(**a**) Semivariograms computed with data collected for both DTS (Distributed Temperature Sending) units. Data were integrated over the entire experimental time. The estimation of the best combination of range and nugget (R^2^ > 0.95) allows for fitting a Gaussian model on experimental data. (**b**) Effect of the integration time on the determination of the semivariogram. The data considered in this example are data from the Ultima S DTS collected in the warm calibration bath. Semivariograms were first constructed for each 20 s sampling times (grey lines), then constructed considering different cases—10 min integration time (blue lines), 1 h integration time (red lines), and 5 h integration time (black line). (**c**) Variability of semivariograms estimated for each integration time over lag distance.

Figure 6a shows semivariograms computed from data collected in the warm calibration bath. Once again, semivariograms were also constructed from cold bath data, but results are not presented here given the similarity of the results in both calibration baths. Figure 6a clearly highlights that the correlation length is larger for measurements collected with the XT DTS unit than for measurements collected with the Ultima S DTS unit. The Gaussian model fits perfectly with experimental data.

The range α estimated for the XT DTS measurements in the warm calibration bath equals 78 cm (with standard deviation of 7.49 cm), while the range estimated for the Ultima S DTS measurements is 29 cm (with standard deviation of 1.01 cm). In other words, the correlation length is more than two times lower for data collected with the Ultima S DTS unit. Concerning the nugget effect, its value was evaluated as 35% of the variance (with standard deviation of 21%) for both units. Since the value of the variance is 0.0028 °C^2^ for the XT DTS measurements and 0.0061 °C^2^ for the Ultima S DTS measurements, the data noise of each DTS unit can be, respectively, evaluated around 0.031 and 0.046 °C.

Semivariograms were computed considering different integration times, as illustrated in Figure 6b. First, semivariance was calculated for each sampling interval (20 s). Then, moving averages were calculated considering different time intervals (10 min, 1 h, and 5 h). In each case, the standard deviation of semivariance was calculated over lag distance. Results are shown in Figure 6c. Broadly speaking, the integration time does not affect the estimation of correlation length, since the global shape of the semivariogram remains similar in each case (Figure 6b). However, the results are much more variable when semivariance is calculated from the shortest interval time (20 s), as shown in Figure 6c. As a result, the analysis of a single isolated semivariogram could lead to the conclusion that its range varies between 0.15 and 1.35 m. The correlation length could; therefore, be largely over- or underestimated. As the data interval is made larger, the variability of semivariance decreases significantly. Integrating data over a 10 min interval time leads to dividing by four the variability of the semivariance. For a 1 h integration time, the mean variability of semivariance is 0.01, meaning that the correlation length can be much more accurately estimated.

#### 3.2.3. Using the Derivative Method

The estimation of the spatial resolution of both DTS units from the “90% step change” method suggests that cable section lengths in the sandbox are equal or larger than the spatial resolution of the units. However, this argument does not seem strong enough to validate measurements considering the high variability of temperature along the cable, and especially within the different cable sections. Thus, the derivative method was applied along the temperature profile. 

**Figure 7:**
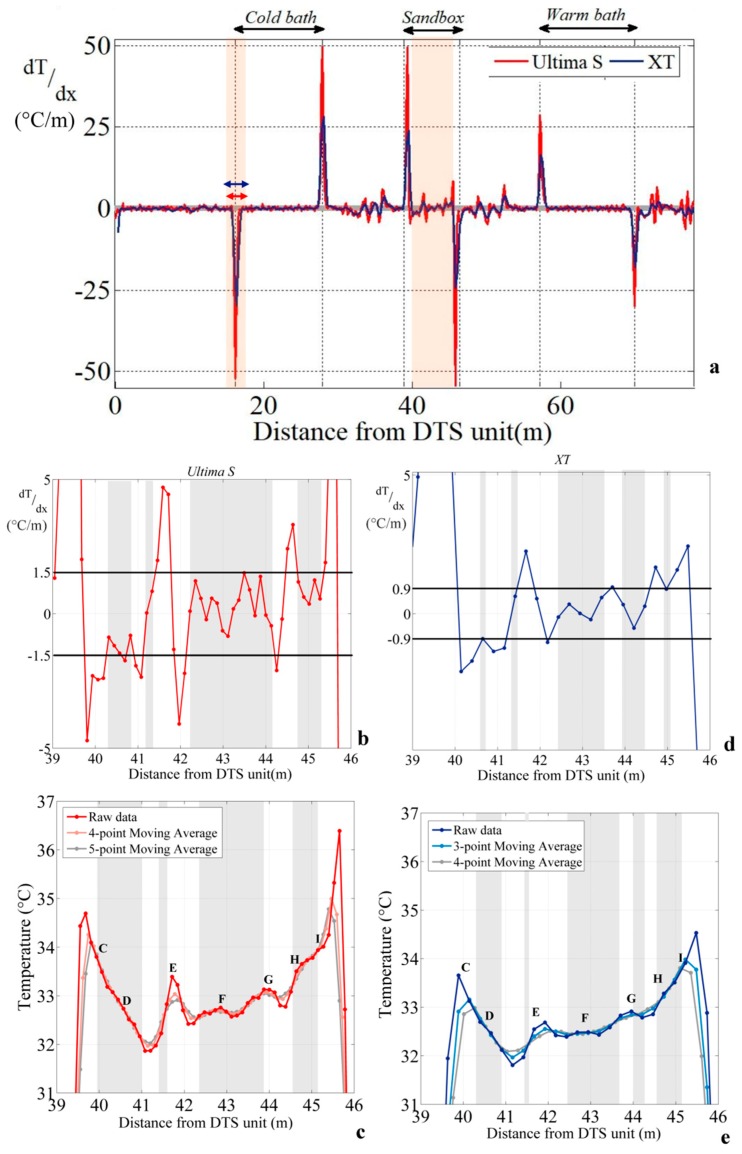
(**a**) Derivative of the temperature with respect to distance. Data were collected during the first heating period, at 02:02:14 p.m. for the Ultima S DTS (Distributed Temperature Sensing) unit and at 02:02:01 p.m. for the XT DTS unit. (**b**) Focus on the derivative of the temperature with respect to distance calculated in the sandbox for the Ultima S DTS unit. Horizontal grey bands represent significant measurement sections defined when the derivative ranges between −1.5 and 1.5 °C/m. (**c**) Significant measurements points for data collected with the Ultima S DTS unit. (**d**) Focus on the derivative of the temperature with respect to distance calculated in the sandbox for the XT DTS unit. Horizontal grey bands represent significant measurement sections defined when the derivative ranges between −0.9 and 0.9 °C/m. (**e**) Definition of significant measurements points for data collected with the XT DTS unit.

Figure 7a shows the evolution of the derivative of the temperature with respect to distance. First of all, the derivative is close to zero when temperature variations are smooth (external sections) or when temperature is constant over a certain length (e.g., inside calibration baths). Because of noise measurements, the derivative ranges between −1.5 and 1.5 °C/m for data collected with the Ultima S DTS unit and between −0.9 and 0.9 °C/m with the XT DTS unit along such sections. This suggests that DTS measurements collected with DTS units in these sections are fully representative of the effective average temperature and that temperature changes, especially in external sections, occur at a scale greater than the spatial resolution.

Conversely, Figure 7a shows that the derivative varies greatly when a step change in temperature occurs along the cable. This is the case, for instance, at the inlet and the outlet of calibration baths. The derivative reaches ±50 °C/m and ±28 °C/m for the cold bath and the warm bath, respectively, for data collected with the Ultima S DTS unit. For the same baths, it reaches ±27 °C/m and ±18 °C/m, respectively, for data collected with the XT DTS unit. The amplitude of the derivative is twice larger for the Ultima S DTS unit than for the XT DTS unit. This difference is due to the coarser spatial resolution of the XT DTS unit, which smoothes temperature measurements. As highlighted previously when using the DTS temperature data model, in case of a step change, the distance over which the derivative varies from zero is twice the length of spatial resolution. Here, this length is 1.02 m for the Ultima S DTS unit and 1.52 m for the XT DTS at the inlet of the cold bath, meaning that the spatial resolution can be estimated at 76 cm for the XT DTS unit and at 51 cm for the Ultima S DTS unit. These values are consistent with the spatial resolution estimated for each DTS unit from the “90% step change” method, confirming that the effective spatial resolution can be also simply estimated or confirmed by the analysis of the derivative of the temperature profile.

The analysis of the derivative provides also an estimate of the representativeness of measurements along the cable. As shown in Figure 7a, local changes of temperature during heating period due to electrical connections induce localized changes in the derivative at the inlet and outlet of the heated section (±50 °C/m for the Ultima S DTS unit and ±25 °C/m for the XT DTS unit). Elsewhere in the sandbox, one can define sections where the variations of the derivative are limited within a narrow range. Concerning data collected with the Ultima S DTS unit, Figure 7c highlights cable sections where the derivative fluctuates between ±1.5 °C/m (grey bands). If we assume now that, in these sections, measurements are representative of the effective temperature during heating periods, this leads to validate temperature measurements all along sections CD, FG, and HI. Concerning both sections DE and EF, local deviations of the derivative indicates that some measurements are not fully representative of the effective temperature, meaning that temperature variations occur at a scale smaller than the spatial resolution of the DTS unit. This conclusion should be considered for the data interpretation. However, at least five measurement points seem to be valid along these sections and could; therefore, be directly interpreted. Lastly, section GH should be entirely excluded from the data interpretation. It is probably heat propagation from section FG that affects, unequally, section GH according to the distance between both sections. The same conclusions can be drawn by calculating moving averages according to the spatial resolution of the DTS units, as shown in Figure 7c. Sections where the data averaging does not affect temperature profiles correspond to sections where the values of derivatives are low enough to validate the data. This suggests that, in such cable sections, successive measurement points could be independently interpreted at the sampling interval. 

The same approach was applied for data collected with the XT DTS unit, as shown in Figure 7d,e. Results suggest also that temperature measurements can be validated between 42.5 and 43.5 m (i.e., for some measurement points along sections EF and FG). Elsewhere, the validation of data is much tougher because of the larger sampling interval and spatial resolution. Indeed, some measurements seem to be valid along the heated section (40.8, 41.4, 44.1–44.2, and 45 m), but these correspond to a limited number of measurement points (one or two in each portion).

## 4. Discussion

### 4.1. Comparison of the Methods for Estimating the Effective Spatial Resolution

Three approaches were presented to estimate the effective spatial resolution of each DTS unit: The “90% step change” method, the estimation of the correlation length, and the derivative method. Table 1 summarizes the results obtained for each method and allows for comparison with DTS units’ specifications provided by the manufacturer. 

As expected, the Ultima S DTS unit has a finer spatial resolution than the XT DTS unit. The application of the “90 % step change” method and the application of the derivative method in calibration baths provide almost the same estimation of the effective spatial resolutions. These estimates are one and half to two times larger than DTS units’ specifications provided by the manufacturer, which is consistent considering the difference when evaluating this parameter (method, conditions, cable). Although the effective spatial resolution is larger than specified, these values remain much more reasonable than demonstrated in some other studies [15,17], which is promising in the deployment of FO-DTS in laboratory tests.

The construction of experimental semivariograms provides complementary insights about DTS measurements by estimating both the correlation length of measurements and the data noise. For both DTS units and both methods, the effective spatial resolution is larger than the correlation length. However, estimated correlation lengths are close to the value of spatial resolution provided by the manufacturer. Practically, the correlation lengths were estimated with data collected inside calibration baths, while the effective spatial resolution was estimating using sharp step changes in temperature along the cable. Considering the high thermal conductivity of the cable (induced by the steel armored protection), conductive heat transfer should occur along the cable because of the temperature gradient existing between calibration baths and external sections.

According to the Fourier’s law of heat conduction, the rate of transfer is a function of the temperature gradient and the properties (thermal conductivity and section) of the cable which conducts heat. Although most of the heat is lost by advection and conduction perpendicularly to the FO cable, lateral heat conduction along the cable cannot be considered as negligible. Knowing the thermal conductivity of the steel, the significance of lateral effect of heat conduction (along the cable) was verified by modelling lateral variations of temperatures, induced by a sharp step change of temperature, by solving the 1D heat conduction equation using the finite difference method [34]. In this simple case, a 20 °C temperature gradient along the cable induces significant temperature changes in the first 50 cm distance from the step change that can reach a few degrees in 5 h.

Thus, the difference between the effective spatial resolution and the correlation length could certainly be attributed to heat conduction occurring along the fiber-optic cable towards step changes in temperature. This would also explain why the estimated values for the effective spatial resolution and the correlation lengths differ in practice while both estimates are similar in the model (which does not consider heat conduction). Thus, our results suggest that the correlation length provides an estimate of spatial resolution close to the manufacturer’s values, while the derivative method and the “90% step change” method provide an estimate that takes into account both the capabilities of the units and the effect of fiber-optic cable, such as heat conduction that occurs during experiments, especially close to abrupt temperature step changes.

The derivative method seems to be a simple and powerful tool for characterizing the quality and the representativeness of data all along the cable. It highlights the effect of spatial resolution on measurements all along the cable, and demonstrates the ability of each DTS unit to measure the variability of temperature along the cable. The approach provides a direct and simple detection and localization of sections where data measurements may not be representative of temperature changes. Conversely, it allows for validating measurements along some sections of the cable by defining sections where measurement points seem to be representative. Along these sections, data can be considered as representative of the effective temperature averaged over the length of the spatial resolution. It should be noted that the derivative can be estimated interchangeably using a forward (Equation (3)) or a backward difference. It just should be specified for the data interpretation. 

Both the step change and the derivative methods require the use of at least one calibration bath in order to ensure the presence of a sharp step change in temperature along the cable, surrounded by long enough sections of cable maintained at a constant temperature. Conversely, the correlation length method can be applied without the existence of a temperature step change, with the advantage of defining spatial resolution independently of conductive heat transfer along the cable. It only requires deploying long enough sections of cable in homogeneous and constant temperature areas (e.g., in baths maintained at ambient temperature). The main advantage of the derivative method is the direct evaluation of the quality of the measurements all along the cable independently of the presence of calibration baths. The use of this method during field applications can be particularly, interesting since experimental conditions are often highly different of ideal laboratory conditions and since the use of calibration baths is not always feasible. 

### 4.2. Feasibility of Using FO-DTS in Laboratory Tests

The comparison of temperature measurements, recorded with both DTS units, shows that both provide an excellent measurement of temperature over time. Relative uncertainty of temperature measurement can be estimated at 0.03 °C and absolute uncertainty of measurement can be estimated at 0.15 °C for the calibration considered here. The quality of data is independent of the sampling interval and the high performance of the internal calibration of both devices in double-ended configuration provides high-quality data. It can be concluded that both DTS units’ performances are quite similar where temperature is uniform along the cable (calibration baths for instance) or where temperature changes are smooth (e.g., in some external sections or in the sandbox during recovery periods). Along these sections, DTS devices can be used interchangeably and the sampling interval and the spatial resolution do not affect the ability of temperature measurements.

The cable section lengths in the sandbox vary between 0.9 and 1 m, meaning that cable section lengths in the sandbox are equal or larger than the effective spatial resolution of units. Thus, the dimensions of the sandbox seem adapted to achieve DTS measurements with such DTS devices. However, seeing the high temperature variability along the heated section, the application of the derivative method is the most efficient method to evaluate the representativeness of measurement along the cable. Obviously, the Ultima S DTS should be preferred for measurements in the sandbox. Because of its improved spatial resolution, it provides larger sections of significant data. Only one cable section should be disregarded for data interpretation. Elsewhere, the number of measurement points with respect to spatial resolution is great enough on each section to interpret data. 

## 5. Conclusions

We showed that the estimation of the effective spatial resolution of DTS data during an experiment depends on the technical specifications of the DTS unit but also on the methods used that may be more or less sensitive to fiber-optic cable properties. Thus, the estimation of the correlation length in calibration baths provides an estimation of the effective spatial resolution relatively close to the technical specifications of DTS units provided by the manufacturer. In the field or during lab experiments, the estimation of the effective spatial resolution defined from a step change is also affected by heat conduction that may increase the length of the step change according to the thermal conductivity of the cable and to the temperature gradient. The application of the “90% step change” method in the field provides an effective spatial resolution that should not necessarily be extrapolated to the whole cable length, since the rate of conductive transfer varies along the cable according to local temperature gradients. This point should be considered cautiously during active-DTS experiments, where temperature gradients may greatly vary both in time and space. Note, also, that this point may explain the discrepancies, generally highlighted in literature, between the values of spatial resolution estimated from the field and the ones provided by the manufacturers, even when the same method is used to estimate the spatial resolution [15]. 

We also showed that the derivative method is a simple and powerful tool for characterizing the quality and the representativeness of data all along the cable. The application of the method permits the validation of measurements by defining cable sections where measurement points are representative of the effective temperature averaged over the length of the spatial resolution. 

To summarize, we demonstrated the feasibility of conducting DTS measurements during laboratory experiments. It requires keeping in mind that spatial resolution fully controls the ability of interpreting DTS measurements, and that DTS devices with the best spatial resolution possible should be preferred during lab experiments. Lastly, the modelling of DTS data could be improved and used to reproduce temperature profiles during experiments, knowing the effective spatial resolution of the DTS units and the data noise of measurements. In further applications, whether during laboratory tests or during field experiments, the issue of the meaning of DTS measurements could be raised beforehand through the data modelling that could strengthen DTS applications, especially in heterogeneous environments. An inverse model could also be proposed and developed to model the efficient temperature along the cable. This will be the subject of future works.

## Figures and Tables

**Table 1:** Comparison of effective spatial resolutions estimated from the different methods for both DTS units.

		Ultima S DTS Unit	XT DTS Unit
Spatial Resolution Provided by the Manufacturer		33 cm	54 cm
“90% step change” method	Effective spatial resolution [m]	52 to 67 cm	84 to 90 cm
Derivative method	Effective spatial resolution [m]	51 to 57 cm	76 to 78 cm
Analysis of the semivariograms	Correlation length (σ [cm])	29 to 31 cm	66 to 78 cm
	Data noise [°C]	0.046	0.031
